# Antineuroinflammatory and neurotrophic effects of CNTF and C16 peptide in an acute experimental autoimmune encephalomyelitis rat model

**DOI:** 10.3389/fnana.2013.00044

**Published:** 2013-12-30

**Authors:** Marong Fang, DaQiang He, Fan Zhang, Zhiying Hu, Jing Yang, Hong Jiang, Shu Han

**Affiliations:** ^1^Institute of Neuroscience, Zhejiang University School of MedicineHangzhou, China; ^2^Department of Obstetrics and Gyneocology, Hangzhou Red Cross HospitalHangzhou, China

**Keywords:** multiple sclerosis, anti-inflammatory, demyelination, neuroprotective effects

## Abstract

Experimentalallergic encephalomyelitis (EAE) is an animal model for inflammatory demyelinating autoimmune disease, i.e., multiple sclerosis (MS). In the present study, we investigated the antineuroinflammatory/neuroprotective effects of C16, an ανβ3 integrin-binding peptide, and recombinant rat ciliary neurotrophic factor (CNTF), a cytokine that was originally identified as a survival factor for neurons, in an acute rodent EAE model. In this model, C16 peptide was injected intravenously every day for 2 weeks, and CNTF was delivered into the cerebral ventricles with Alzet miniosmotic pumps. Disease severity was assessed weekly using a scale ranging from 0 to 5. Multiple histological and molecular biological assays were employed to assess inflammation, axonal loss, neuronal apoptosis, white matter demyelination, and gliosis in the brain and spinal cord of different groups. Our results showed that the EAE induced rats revealed a significant increase in inflammatory cells infiltration, while C16 treatment could inhibit the infiltration of leukocytes and macrophages down to 2/3–1/3 of vehicle treated EAE control (*P* < 0.05). The delayed onset of disease, reduced clinical score (*P* < 0.01) in peak stage and more rapid recovery also were achieved in C16 treated group. Besides impairing inflammation, CNTF treatment also exerted direct neuroprotective effects, decreasing demyelination and axon loss score (*P* < 0.05 versus vehicle treated EAE control), and reducing the neuronal death from 40 to 50% to 10 to 20% (*P* < 0.05). Both treatments suppressed the expression of cytokine tumor necrosis factor-α and interferon-γ when compared with the vehicle control (*P* < 0.05). Combined treatment with C16 and CNTF produced more obvious functional recovery and neuroprotective effects than individually treatment (*P* < 0.05). These results suggested that combination treatment with C16 and CNTF, which target different neuroprotection pathways, may be an effective therapeutic alternative to traditional therapy.

## INTRODUCTION

Multiple sclerosis (MS) is a central nervous system (CNS) autoimmune disease with symptoms that include neurological impairment and motor deficits (Hou et al., 2012). MS results from attack of myelin by the immune system, which leads to axonal and neuronal degeneration (Stanislaus et al., 2004; Huizinga et al., 2008; Yin et al., 2010). Experimental autoimmune encephalomyelitis (EAE) is the primary animal model of MS, characterized by microglial activation and lymphocyte infiltration (Arnon and Aharoni, 2009; Mix et al., 2010; Yin et al., 2012). Consequent demyelination, axonal injury, and neuronal loss also underlie the disability and disease progression observed in EAE (Arnon and Aharoni, 2009).

Current treatment strategies for EAE and MS mainly target injury sites at the CNS, interfering with both neuroinflammation and neurodegeneration (Arnon and Aharoni, 2009). To migrate into sites of inflammation, leukocytes first tether and roll along the vessel, then adhere and emigrate out of the vasculature (Alon and Luscinskas, 2004). Leukocyte-endothelium interactions have been recognized as playing an important role in this leukocyte infiltration process (Weerasinghe et al., 1998; Alon and Luscinskas, 2004). Additionally, previous studies have shown that integrin alpha V beta 3 (ανβ3) can modulate leukocyte adhesion to intercellular adhesion molecule-1 (ICAM-1) and enable leukocytes to migrate effectively across the endothelium (Weerasinghe et al., 1998).

The synthetic C16 peptide, representing a functional laminin domain, selectively binds to ανβ3 integrin (Ponce et al., 1999). Since ανβ3 has been shown to mediate leukocyte adhesion and migration, this ανβ3-binding peptide could block ανβ3 and reduce monocyte transmigration across the endothelial cell layer. In fact, C16 exerted blocking effects on ανβ3 that were similar to the effects of blocking antibodies against ανβ3 integrins *in vitro* (Han et al., 2010; Fang et al., 2013). Intravenous injection of C16 has also been shown to reduce the infiltration of leukocytes, extravasation of macrophages, and accumulation of activated microglia *in vivo* (Han et al., 2010; Fang et al., 2013). The fact that leukocyte infiltration was inhibited with no effects on systemic leukocyte counts implies that the C16 peptide may be a potential therapeutic agent for MS treatment (Han et al., 2010).

Ciliary neurotrophic factor (CNTF) was described originally as a neurocytokine that could promote the survival of neurons and the differentiation and maturation of oligodendrocytes (Linker et al., 2002; Stankoff et al., 2002; Kuhlmann et al., 2006). As a modulator in neuroinflammation and a neurotrophic agent, CNTF has also recently been proposed to be a major protective factor in the treatment of inflammatory demyelinating disease (Linker et al., 2002; Kuhlmann et al., 2006). Indeed, clinical disabilities in acute EAE are due in part to demyelination and axonal disruption, which are caused by an early inflammatory attack on white matter (Brück, 2005). Demyelination in the peripheral nerve system (PNS) and CNS is an important cause of the neurological deficits in acute EAE induced by whole spinal cord homogenates (Pender, 1989). Thus, CNTF may be a useful clinical agent.

C16 has been shown to reduce inflammatory cell infiltration in EAE (Fang et al., 2013). Moreover, since previous studies have suggested that promoting remyelination appeared to be a crucial therapeutic challenge of MS, CNTF, as a survival and differentiation factor for neurons and oligodendrocytes, may promote remyelination and prevent neuronal damage. Combination of different drugs that target different pathways of neuroprotection may produce more effective therapeutic results; therefore, the present study was designed to investigate the combined therapeutic effects of C16 and CNTF in an acute EAE model induced in Lewis rats, and in this study, combination of different drugs that target different pathways of neuroprotection may produce more effective therapeutic results; therefore, the present study was designed to investigate the combined therapeutic effects of C16 and CNTF in an acute EAE model induced in Lewis rats.

Among a variety of EAE models in rodents, the acute EAE model induced in Lewis rats is a well-established model of MS, characterized by a single peak of paralysis after which animals recover spontaneously (Almolda et al., 2011). The utilization of this model gives us an opportunity to elucidate the induction, peak, and resolution of the inflammation-based immune response of MS. However, the most obvious shortcoming of this acute model was not allow us to test the impact of C16 on advanced manifestations of the MS disease, including the long-term occurrence of extensive axonal damage resulting from sustained demyelination (Basso et al., 2008; Soulika et al., 2009; Berard et al., 2010), thus the EAE process were observed within a period of 8 weeks.

## MATERIALS AND METHODS

### EXPERIMENTAL PROCEDURES

#### Animals and EAE induction

A total of 69 adult male Lewis rats, weighing 250–300 g, were obtained from the Laboratory Animal Services Centre of the Zhejiang University. Of these, five were used as the normal control group and the remaining 64 were randomly assigned into four groups: the vehicle control treated group, C16 [synthesized by Shanghai Science Peptide Biological Technology Co., Ltd, China; 1 mg/per day, intravenous (IV) injection] treated group, CNTF [PeproTech Inc., NJ, USA; 10 μg, intrathecal administration (IT), see details below] treated group, and CNTF + C16 treated group. EAE was induced in both drug- and vehicle-treated rats with subcutaneously nuchal injection of 0.2 mL a 1:1 mixture of guinea pig spinal cord homogenate (GPSCH) and complete Freud adjuvant (CFA), containing 0.5 mg of heat killed *Mycobacterium tuberculosis* (Difco Laboratories, Detroit, MI, USA). The rats in the normal control group were injected with CFA emulsified 1:1 with 0.9% saline. Immediately thereafter, and again 48 h later, the rats received an intraperitoneal injection of 300 ng Pertussis toxin per animal (Sigma-Aldrich, St. Louis, MO, USA).

Beginning on day 7, the animals were weighed and assessed for clinical signs of disease on a daily basis. Disease severity was assessed using a scale ranging from 0 to 5: grade 0 = no signs, grade 1 = partial loss of tail tonicity, grade 2 = loss of tail tonicity, grade 3 = unsteady gait and mild paralysis, grade 4 = hind limb paralysis and incontinence, and grade 5 = moribund or death (Yin et al., 2010). The EAE model was generally considered a success when the score exceeded 2, and scoring of the animals was continued until the time of sacrifice.

All animal procedures used in this study were carried out in accordance with the National Institute of Health Guide for the Care and Use of Laboratory Animals.

### PREPARATION OF OSMOTIC PUMPS AND DRUG DELIVERY

Ciliary neurotrophic factor and vehicle were delivered continuously via implanted Alzet osmotic minipumps over 2 weeks (pump model 2002; 0.5 μL/h). All the osmotic minipumps were implanted immediately after receiving EAE induction (GPSCH and first time Pertussis toxin injection). Before implantation, 48 osmotic minipumps were prepared under sterile conditions and filled with PBS (*n* = 16 for the vehicle-treated group) and CNTF in saline (*n* = 16 rats per group for CNTF- and CNTF+C16-treated groups). Cannula consisting of polyurethane tubing were sterilized overnight in 100% ethanol before being attached to the flow moderator of the pump. The pumps were incubated overnight at room temperature in sterile saline for priming. The animals were anesthetized with intraperitoneal injections of 1% Nembutal (40 mg/kg), under an operating microscope, the tabs on the top of the cannula were attached to the electrode holder of the stereotaxic apparatus, and the catheters were introduced into the cerebral ventricle through small holes in the skull, according to the manufacturer’s instructions for the Alzet brain infusion kit (The surgical details of placing Alzet brain infusion kit could be found at http://www.alzet.com/products/brain_infusion_kit/how_it_works.html). The infusion was started immediately. The anchoring screws were set with dental cement, and the pump was placed in a subcutaneous pocket. The scalp wound was closed in layers. After the surgery, rats were placed in temperature- and humidity-controlled chambers overnight, and penicillin was injected intramuscularly (25,000 UI per rat).

### INTRAVENOUS INJECTION OF C16

C16 peptide was dissolved in distilled water with 0.3% acetic acid. The peptide solution was sterilized through a 0.22-μm disk filter and neutralized with NaOH. This solution was buffered by adding an equal volume of sterile PBS, and the final concentration of C16 was 2 mg/mL. The vehicle solution was prepared in the same manner (0.3% acetic acid neutralized to pH7.4 with NaOH, then buffered by adding an equal volume of sterile phosphate-buffered saline) without adding the peptide. Rats in the C16-treated group (*n* = 16) each received 0.5 mL of C16 solution, while the vehicle (control) group was treated with 0.5 mL of the same solvent without adding the peptide via IV injection of the tail vein. The first dose was given immediately after EAE induction (see above), and the IV injection repeating every day for 2 weeks in the tail veins was achieved by starting injections at the caudal end of the base of the tail and into one vein. Injection sites were moved to the alternate left or right sides and increasingly rostral on subsequent days.

### PERFUSION AND TISSUE PROCESSING

Animals from each group were sacrificed after 2 or 8 weeks (five rats per time point in each group) post-immunization. Rats were anesthetized with intraperitoneal injections of 1% Nembutal (40 mg/kg) and perfused intracardially with cold saline followed by 4% paraformaldehyde in 0.1 M phosphate buffer (PB, pH 7.4). After undergoing *ex vivo* magnetic resonance imaging (MRI) scans, the spinal cord and brain tissues were carefully dissected. One centimeter of the lumbar spinal cord and a half of the brain of each animal were fixed in the same fixative for 4 h and then transferred into 30% sucrose in PBS until the tissue sunk to the bottom of the container. Twenty micro-thick sections were cut on a freezing microtome through the coronal plane of the brain and transverse plane of the spinal cord using a Leica cryostat and then mounted onto 0.02% poly-L-lysine-coated slides. All sections were collected for histological assessment and immunohistological and immunofluorescent staining. The remains of the CNS tissue were fixed in 2.5% glutaraldehyde solution and examined by transmission electron microscope.

### MAGNETIC RESONANCE IMAGING

*Ex vivo* MRI scanning of fixed brains was performed using a clinical 3.0 T MRI machine (Signa HDxt 3 T) equipped with a dedicated solenoid rat coil. Rats were placed in the cradle supine position. T2-weighted sequences (15 contiguous coronal slices of 1.5 mm each) were collected with the following characteristics: TE = 120 ms, TR = 3200 ms, slice thickness = 1.5 mm, number of slices = 15, 320 × 192 matrix, field of view = 60 mm × 60 mm, flip angle = 90°.

### HISTOLOGY ASSESSMENT

Hematoxylin and eosin (H&E) staining and cresyl violet (Nissl) staining were employed to assess inflammation and neuron survival, respectively. Neuron counts from both spinal cord anterior horns were performed and restricted to the neurons with a well-defined nucleolus and a cell body with adequate amounts of endoplasmic reticulum. Digital images were collected using a Nikon TE-300 microscope in three visual fields/per section with 200× magnification under bright field viewing. Besides, Bielschowsky silver staining was performed as previously described to estimate axonal loss (Fang et al., 2013) and MBP immunohistochemical labeling was used to evaluate the degree of demyelination.

### QUANTITATIVE MEASUREMENTS

Images of were taken on an Nikon brightfield and fluorescence microscope and images digitized with an attached Spot camera (MI, USA). All images within a type of staining were taken with the same exposure time. Quantification of myelinated area was performed by calculation of MBP-positive pixels in one series of sections (200 μm apart) by the threshold feature of Scion Image software (Scion Corporation, Frederick, MD, USA). Briefly, after the transformation of each section to a binary image, the percentage of black pixels was calculated for every section, and the average of three consecutive images was calculated. Since single inflammatory cells were too difficult to discern at the injury site, the infiltration of inflammatory cells was digitized using a 5× objective and the area occupied by these extravasated cells were calculated for the three sections (five fields in each). To provide a measure of the number of preserved axons, the number of Bielschowsky silver stained axons intersecting 100-mm spaced horizontal (five) and vertical (six) lines were counted in each image through a series of sections (each 200 μm apart; Han et al., 2010; Muradov and Hagg, 2013) under 200× magnification.

### IMMUNOHISTOCHEMICAL STAINING

Slides were warmed for 20 min on a slide warmer, and a ring of wax was applied around the sections with a PAP pen (Invitrogen, Carlsbad, CA, USA). After rinsing in 0.01 M Tris-buffered saline (TBS) for 10 min, the sections were permeabilized and blocked with 0.3% Triton X-100/10% normal goat serum in 0.01 M PBS for 30 min, then incubated with the following polyclonal rabbit antibodies: anti-CD4 (1:500, AbCam, Cambridge, MA, USA), anti-CD45 (1:200; AbCam), CD68/ED1 (1:200; Santa Cruz Biotechnology, Santa Cruz, CA, USA), anti-tumor necrosis factor alpha (TNF-α; 1:1000; ProSci Incorporated, CA, USA), anti-caspase 3 (1:500; Cayman Chemical, Ann Arbor, MI, USA), and anti-160KD neurofilament M (NF-M; 1:1000; Neuromics, MN, USA), as well as mouse anti-myelin basic protein (MBP; 1:500; AbCam) overnight at 4°C. The process of immunohistochemical staining was performed as described previously (Fang et al., 2013). Primary antibody omission controls were used in order to confirm further the specificity of the immunohistochemical labeling. Five sections from the motor cortex and anterior horns of the spinal cord of each animal were randomly selected, and images were photographed under 200× magnification in three visual fields per section.

### IMMUNOFLUORESCENCE STAINING

Sections were pretreated with the same method described above, incubated with polyclonal rabbit anti-glia fibrillary acidic protein (GFAP; 1:200; Thermo Fisher Scientific, Waltham, MA, USA) overnight at 4°C, then washed with PBS and incubated with FITC-conjugated goat anti-rabbit IgG secondary antibodies (1:200 dilution) for 1 h at 37°C (Invitrogen). The sections were mounted with antifade Gel/Mount aqueous mounting media (SouthernBiotech, AL, USA). All control sections were incubated in PBS without primary antibodies.

### PROCESSING FOR ELECTRON MICROSCOPY

Tissues fixed with 2.5% glutaraldehyde were washed three times with 0.1 M PB, post-fixed in 1% osmium tetroxide at 4°C overnight, and then washed three times with 0.1 M PB. The processing for electron microscopy was performed as described previously (Fang et al., 2013). Images were captured first at low resolution and then at higher magnification in different regions of the white matter.

### CYTOKINE QUANTIFICATION BY ENZYME-LINKED IMMUNOSORBENT ASSAY

Peripheral blood samples were collected from rats that were sacrificed by decapitation at weeks 2 and 8 post-immunization (*n* = 3 per time point for each group). Plasma samples were collected on ice following centrifuge for 20 min at 1000 × *g* within 30 min of collection and centrifugation at 10,000 × *g* for 10 min at 4°C for complete platelet removal, using heparin as an anticoagulant. All samples were aliquot and stored at -80°C. To assess cytokine expression, plasma samples were incubated in 96-well plates pre-coated with antibodies to IFN-γ (BioLegend Inc., San Diego, CA, USA) and TGF-β (R&D Systems, Minneapolis, MN, USA) at 37°C for 60 min. Horseradish peroxidase (HRP)-conjugated goat anti-rabbit IgG (Bio-Rad, Hercules, CA, USA) diluted 1:2,000 was used as a secondary antibody. The optical density was measured at 450 nm on a Model 680 Microplate Reader (Bio-Rad) and was quantified using GraphPad Prism 4 software (GraphPad Software, Inc, CA, USA).

### WESTERN BLOTTING

Rats were sacrificed by decapitation at weeks 2 and 8 post-immunization (*n* = 3 rats per time point in each group), and whole brain tissues and a 10-mm lumbar spinal cord segment were prepared for Western blotting from each animal. Total proteins were extracted from the intact spinal cord 1 mL EDTA-free ice-cold RIPA buffer with 2 mM PMSF and added protease inhibitor cocktail. Protein concentrations were determined using the Bradford protein assay. SDS-PAGE was performed on 15% polyacrylamide slab gels, and separated proteins were then electrophoretically transferred to PVDF membranes at 70 V for 1.5 h at 4°C in a Bio-Rad TransBlot apparatus. After blocking non-specific binding sites with bovine serum albumin, each membrane was incubated for 12 h at room temperature with primary rabbit polyclonal anti-GFAP (1:500), anti-NF-M (1:2000), anti-TNF-α (1:2000), or anti-caspase 3 (1:500) antibodies. The process of Western blot was performed as previous described (Fang et al., 2011). To normalize protein bands to a gel loading control, membranes were washed in TBST and re-probed with rabbit anti-β-actin antibodies (1:5,000, AbCam) followed by incubation with HRP-conjugated goat anti-rabbit antibodies (1:5,000) and ECL detection. For the negative control, the primary antibody was omitted.

### STATISTICAL ANALYSIS

Kruskal–Wallis non-parametric analysis was used for data presented as percentages. Differences between clinical scores and histological scores were analyzed with Mann–Whitney tests. All data were analyzed in SPSS 13.0 software, and *P*-values less than 0.05 were considered statistically significant. All statistical graphs were created in GraphPad Prism Version 4.0.

## RESULTS

### EFFECTS OF TREATMENT WITH CNTF AND/OR C16 ON PERIVASCULAR/PARENCHYMAL INFLAMMATION IN THE EAE RAT MODEL

**FIGURE 1:**
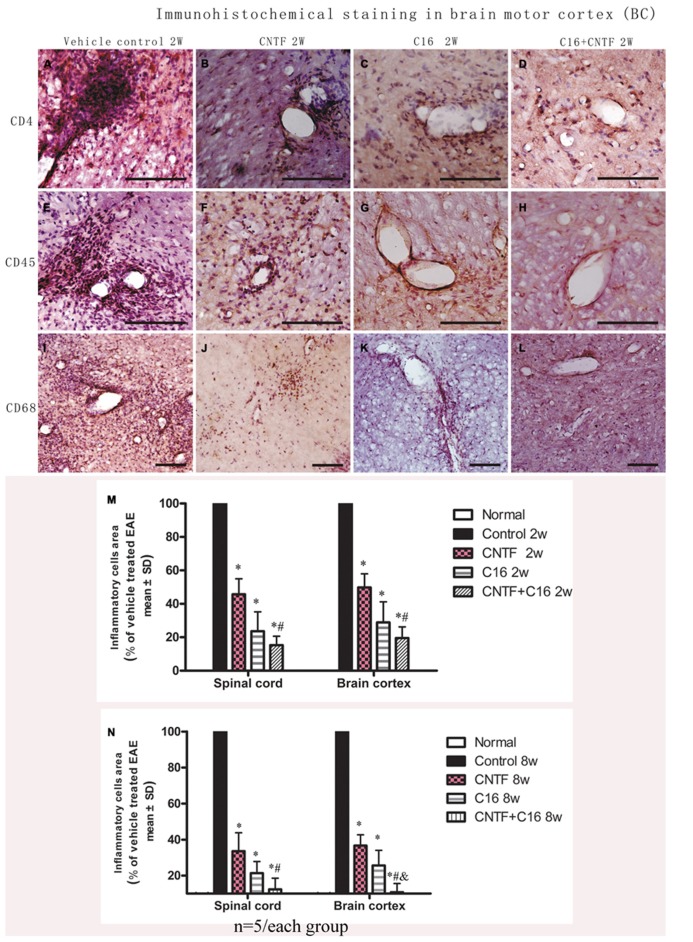
**At week 2 post-immunization, infiltration of inflammatory cells was observed surrounding blood vessels in the cortex and spinal cord of vehicle-treated EAE rats, and this effect was attenuated by treatment with C16 and CNTF. (A–D)** CD4 immunostaining; **(E–H)**, CD45 immunostaining; **(I–L)**, CD68 immunostaining, counterstained with hematoxylin, bar = 100 μm. **(M,N)** CNTF and C16 treatment inhibited inflammatory cell infiltration at week 2 **(M)** and week 8 **(N)** post- immunization. *P < 0.01 versus the vehicle-treated EAE rats; *^#^P* < 0.01 versus CNTF-treated EAE rats; ^&^*P* < 0.01 versus C16-treated EAE rats.

For determination of the types of inflammatory cells infiltrating these tissues, we performed immunostaining for the detection of CD4, a marker for extravasated T lymphocytes; CD45, a pan-leukocyte marker for leukocytes; and CD68, a marker for activated microglia and extravasated macrophages (**Figure 1**). Numerous CD4+, CD45+, and CD68+ inflammatory cells infiltrated the CNS of vehicle-treated EAE rats (**Figures 1A,E,I**), while markedly reduced perivascular and parenchymal infiltrations of these inflammatory cells were detected in CNTF- (**Figures 1B,F,J**), C16- (**Figures 1C,G,K**), and CNTF+C16-treated (**Figures 1D,H,L**) EAE rats.

At the peak time of the disease (2 weeks after immunization), the cerebral cortex and spinal cord of EAE rats treated with vehicle all revealed a significant increase in cellular infiltration. Diffuse infiltration of inflammatory cells appeared not only surrounding blood vessels and under the meninges, but also widely infiltrating the parenchyma of the brain tissue. At week 8 post-immunization, the extent of infiltration and the size of perivascular infiltration foci decreased compared with those at week 2 post-immunization in the vehicle control. Treatment with either C16 or C16 plus CNTF markedly suppressed the infiltration of inflammatory cells in different cell types, but CNTF treatment did not result in obvious reduction of inflammation at week 8 post-immunization. The combined treatment (C16 plus CNTF) caused a more obvious impairment of the inflammatory reaction at week 2 but not at week 8 post-immunization (**Figures 1M,N**).

**FIGURE 2:**
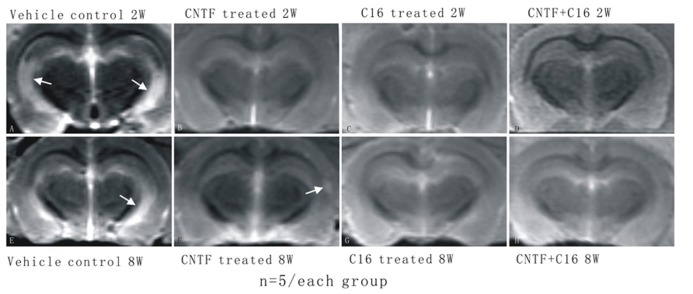
**The MRI T_2_W scanning showed clear hyperintensity lesion site appear in brain cortex of vehicle treated EAE rats but not in CNTF and C16 treated EAE rats.** **(A)** Vehicle treated EAE rats at week 2 post-immunization, arrow illustrating a clear hyperintensity area in the white matter. There were no visible lesion foci in **(B)** CNTF treated EAE rats, **(C)** C16 treated EAE rats, and **(D)** C16+CNTF treated EAE rats at week 2 post-immunization. **(E)** Vehicle treated EAE rats at week 8 post-immunization. Arrow showed hyperintensity lesion area still existed, although a little smaller than lesion site at week 2 post-immunization. An intensity increased lesion area was detected in CNTF treated group [arrow in **(F)**], but not in C16 treated **(G)** and C16+CNTF treated **(H)** EAE rats at week 8 post-immunization.

Magnetic resonance imaging T_2_W scanning revealed clear lesion foci with hyperintensity in the brain tissues of vehicle-treated EAE rats at week 2 post-immunization (white area with high density in **Figure 2A**) and visible lesion still could be found at week 8 post-immunization (density increase area showed by arrow in hippocampi and cortex of vehicle-treated EAE rats in **Figure 2E**) although it smaller than lesion site at week 2 post-immunization. An intensity increased lesion area was detected in CNTF treated group (**Figure 2F**), but not in C16 treated (**Figure 2G**) and C16 plus CNTF treated (**Figure 2H**) EAE rats at week 8 post-immunization. However, there were no visible lesion foci in C16-, CNTF-, and C16 plus CNTF-treated groups at weeks 2 post-immunization (**Figures 2B–D**).

### EFFECTS OF TREATMENT WITH CNTF AND/OR C16 ON DEMYELINATION AND AXON LOSS

**FIGURE 3:**
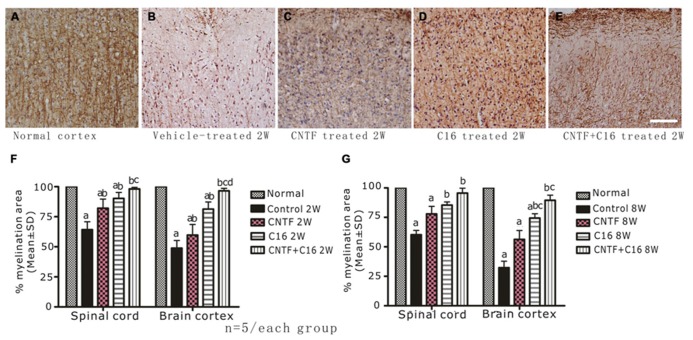
**Treatment with CNTF or C16 reduced demyelination in the spinal cord and cerebral cortex, as revealed by MBP immunostaining, counterstained with hematoxylin (coronal sections of brain motor cortex), Bar = 100 μm.** At week 2 post-immunization, normal control group **(A)**, vehicle-treated EAE rats **(B)**, CNTF-treated EAE rats **(C)**, C16-treated EAE rats **(D)**, and C16+CNTF-treated rats **(E)**. **(F,G)** Treatment with CNTF or C16 prevented demyelination at week 2 **(F)** and week 8 **(G)** post-immunization. *^a^P* < 0.05 versus the normal control; *^b^P* < 0.05 versus vehicle-treated EAE rats; *^c^P* < 0.05 versus CNTF-treated EAE rats; *^d^P* < 0.05 versus C16-treated EAE rats.

Using MBP (one of the major central myelin proteins) immunohistochemical staining, we checked the total demyelination condition of rats in each group. Large plaques of demyelination were detected in the CNS of vehicle-treated EAE rats at the peak time of the disease (week 2 post-immunization, **Figure 3B**), when compared with the normal rats (**Figure 3A**). At week 8 post-immunization, the demyelination condition when compared with the normal rats (**Figures 6A,B**) in the vehicle control was reduced. As expected, the demyelination was dramatically reduced by C16 or CNTF treatment (**Figures 3C–E**), and was reduced even more by combined treatment with C16 plus CNTF when compared with vehicle treatment at both weeks 2 and 8 post-immunization (**Figures 3F,G**).

**FIGURE 4:**
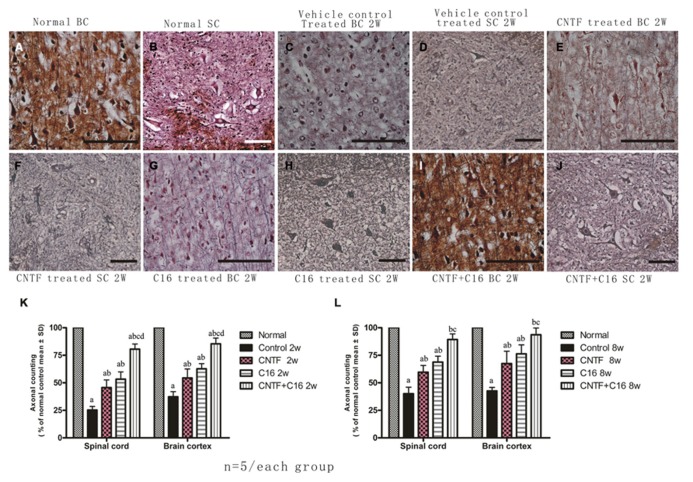
**Treatment with CNTF or C16 alleviated axonal loss in the spinal cord and cerebral cortex, as revealed by Bielschowsky staining, [(A,C,E,G,I): coronal sections of brain motor cortex; (B,D,F,H,J): traverse sections through anterior horn of lumbar spinal cord], bar = 100 μm.** At week 2 post-immunization, **(A,B)** normal control group, **(C,D)** vehicle-treated EAE rats [**(C)** axons were undergoing gradual loss and exhibiting deformed and ovoid formation, the neurons have lost dendrite and axons when compared with the normal neurons in **(A,B)**], **(E,F)** CNTF-treated EAE rats, **(G,H)** C16-treated EAE rats, and **(I,J)** C16+CNTF-treated rats. More axons were shown in these groups. **(K,L)** Treatment with CNTF or C16 prevented axon loss. *^a^P* < 0.05 versus the normal control; *^b^P* < 0.05 versus vehicle-treated EAE rats; *^c^P* < 0.05 versus CNTF-treated EAE rats; *^d^P* < 0.05 versus C16-treated EAE rats.

**FIGURE 5:**
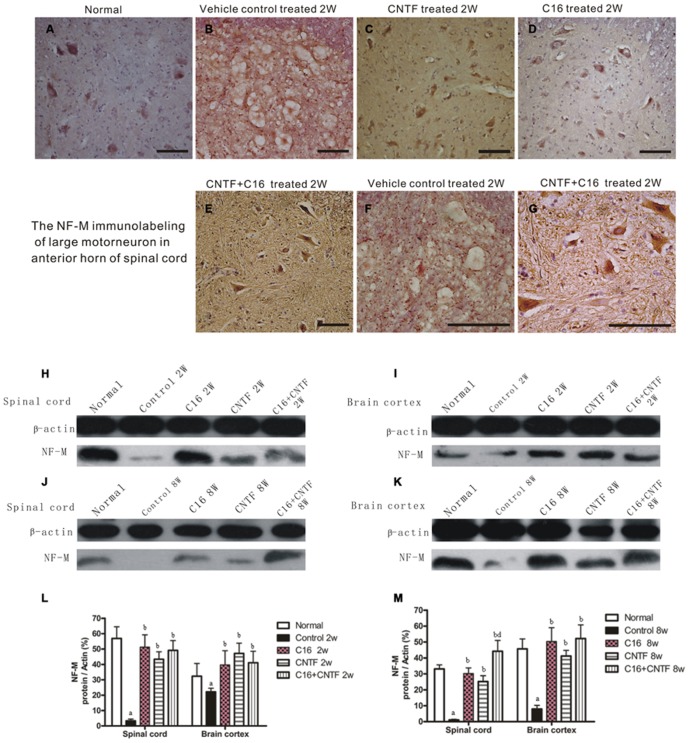
**Treatment with CNTF or C16 alleviated neuronal atrophy and axonal disruption at week 2 post-immunization.** **(A–G)** NF-M immunostaining, counterstained with hematoxylin, traverse sections through anterior horn of lumbar spinal cord, bar = 100 μm. **(A)** Normal control group. **(B)** Inflammatory infiltrates, neuronal atrophy, and dendrite and axonal disruption in vehicle-treated EAE rats. **(C–E)** Lots of perikaryal and axonal hyper-NF-M immunostaining motor neurons appeared in CNTF-treated **(C)**, C16-treated **(D)**, and C16+CNTF-treated **(E)** EAE rats. **(F,G)** The enlarged image of vehicle-treated **(F)** and C16+CNTF-treated **(G)** EAE rats. The motor neurons at the anterior horn of spinal cord lost the normal shape and cell skeleton which labeled by NF-M **(F)**, when compared with the normal shape motor neurons with NF-M labeled kytoplasm and neuritis **(G)**. NF-M expression decreased markedly in both the spinal cord **(H,J)** and cerebral cortex **(I,K)** following EAE induction, but up-regulated after treatment with CNTF or C16, as compared with the vehicle treated control, at weeks 2 and 8 post-immunization, as shown by Western blotting. **(L,M)** NF-M expression in the spinal cord and brain cortex at week 2 **(L)** and week 8 **(M)** post-immunization revealed by western blotting. *^a^P* < 0.05 versus normal control; ^b^*P* < 0.05 versus vehicle-treated EAE rats; *^d^P* < 0.05 versus CNTF-treated EAE rats.

Bielschowsky staining revealed that the injured axons exhibited swelling, deformation, and ovoid formation (**Figure 4C**). Vehicle-treated EAE rats displayed severe axonal loss in both the white and gray matter of the CNS at week 2 post-immunization (**Figures 4C,D**) when compared with the normal rats (**Figures 4A,B**). However, both the C16 and CNTF treatments protected the injured axons and keep them into relatively normal shape (**Figures 4E–L**). In the meantime, immunofluorescence labeling of NF-M, a cytoskeletal intermediate filament protein found specifically in neurons, demonstrated that the lumbar spinal cord neurons were undergoing swelling and disruption, with the dendrites and axons atrophy, shortening, and exhibiting fragmentation, accompanied by the infiltration of inflammatory cells (**Figures 5B,F**). Compared with the vehicle control, more axons with relatively normal formation were maintained in C16-, CNTF-, and C16 plus CNTF-treated EAE rats at each time point (**Figures 5B–G**). Much higher NF-M expression and less severe depletion of motor neurons were also found in C16-, CNTF-, and C16 plus CNTF-treated rats, as compared with the vehicle control at the same time point (**Figures 5F,G**) had showed a typical comparison of injured and protected neurons). Simultaneously, Western blot analysis revealed an obvious decrease in neurofilament M expression in the vehicle control, and this effect was significantly reversed by treatment with C16, CNTF, or C16 plus CNTF at both weeks 2 and 8 post-immunization (**Figure 5**).

**FIGURE 6:**
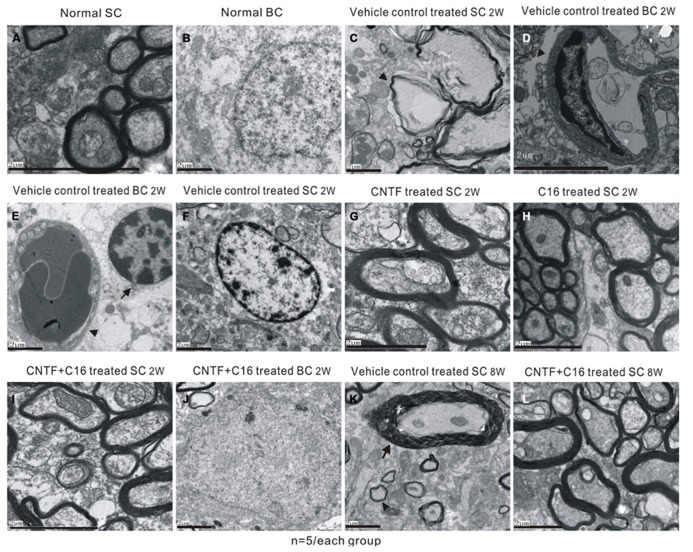
**Electron micrographs demonstrating the prevention of myelination or axon loss and inhibition of neuronal apoptosis by CNTF and C16 treatment.** **(A,B)** Normal control group [**(A)**, normal myelinated axons exhibited dark ring-shaped myelin sheaths surrounding the axon; **(B)**, normal nuclei of neuron with uncondensed chromatin]. **(C–F)** Vehicle-treated EAE rats at week 2 post-immunization. The myelin sheath displayed splitting, vacuoles (showed by arrow), and loose and fused changes, and axons were shrunken and dissolving **(C)**. Severe leaking out of the blood vessels and tissue emeda were detected in the extracellular space surrounding the vessels [showed by arrow in **(D)**]. **(E)** Infiltrated leukocytes (showed by arrow) near the vascular endothelial cell (showed by red arrowhead). **(F)** The neurons of the vehicle control showed apoptotic signs of shrunken nuclei with condensed, fragmented, and marginated nuclear chromatin. The CNTF- **(G)**, C16- **(H)**, and C16+CNTF-treated **(I)** groups exhibited more lightly vacuolated myelin sheaths. The neighboring nuclei **(J)** of C16+CNTF-treated rats resembled the normal ultrastructure. At week 8 post-immunization in the vehicle control group, demylination (arrow) and remyelination (arrowhead) simultaneously appeared in vehicle-treated EAE rats **(K)**. Meanwhile, more remyelinated fibers appeared in C16+CNTF-treated **(L)** groups.

Transmission electron microscopy examination further revealed that a considerable amount of the myelin sheath displayed splitting and vacuolated changes in the vehicle control group (**Figure 6C**) when compared with the normal rats (**Figures 6A,B**). Shrunken axons were covered by disrupted myelin sheaths, and, in some places, the axons even disappeared (**Figure 6C**). Severe edema and leakage of inflammatory cells from the blood vessels were detected in the extracellular space surrounding the vessels (**Figures 6D,E**). The neurons of the vehicle control showed signs of apoptosis, including shrunken nuclei with condensed, fragmented, and marginated nuclear chromatin (**Figure 6F**). In contrast, C16-, CNTF-, and C16 plus CNTF-treated groups exhibited more lightly vacuolated myelin sheaths, with the corresponding axons and neighboring nuclei resembling the normal ultrastructure (**Figures 6G–J**). At week 8 post-immunization, demylination, and remyelination appeared simultaneously in vehicle-treated EAE rats (**Figure 6K**). Meanwhile, more remyelinated fibers appeared in C16-, CNTF-, and C16 plus CNFT-treated groups (**Figure 6L**).

### EFFECTS OF TREATMENT WITH CNTF AND/OR C16 ON APOPTOSIS AND NEURON LOSS IN THE CNS OF EAE RATS

**FIGURE 7:**
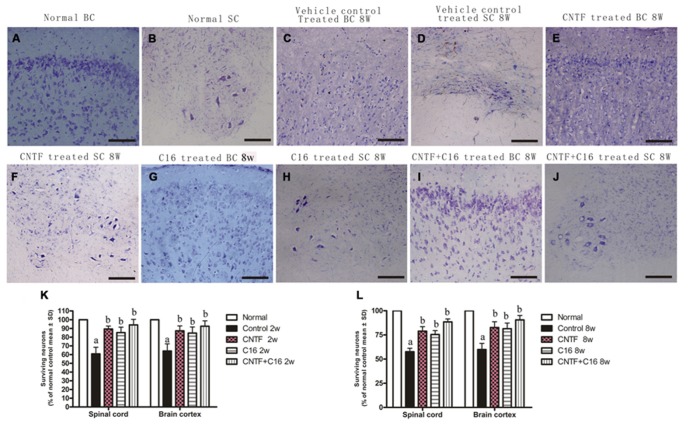
**Treatment with CNTF or C16 reduced the loss of neurons both in the spinal cord and brain.** Nissl staining, bar = 100 μm. [**(A,C,E,G,I)**: coronal sections of brain motor cortex; **(B,D,F,H,J)**: traverse sections through anterior horn of lumbar spinal cord]. At week 8 post-immunization, normal animals **(A,B)**, vehicle-treated EAE rats **(C,D)**, CNTF-treated EAE rats **(E,F)**, C16-treated EAE rats **(G,H)**, and C16 +CNTF-treated EAE rats **(I,J)**. **(K,L)** The CNTF-, C16-, and C16+CNTF- treated EAE rats all gained more neurons in the motor cortex, and anterior horn of the spinal cord. Neuron counts were restricted to the neurons with a well-defined nucleolus and a cell body with adequate amounts of endoplasmic reticulum, in three visual fields/per section with 200× magnification under bright field viewing. Surviving neural cells (each group % of the normal control) in different groups at week 2 **(K)** and week 8 **(L)** post-immunization, calculated after Nissl staining. *^a^P* < 0.05 versus the normal control; *^b^P* < 0.05 versus vehicle-treated EAE rats.

There was marked neuron loss in the anterior horn of spinal cords and motor cortices of the vehicle-treated EAE rats at both weeks 2 and 8 post-immunization (**Figures 7C,D**) when compared with normal animals (**Figures 7A,B**). However, in C16-, CNTF-, and C16 plus CNTF-treated EAE rats, more neurons were present in the CNS (**Figures 7E–L**).

**FIGURE 8:**
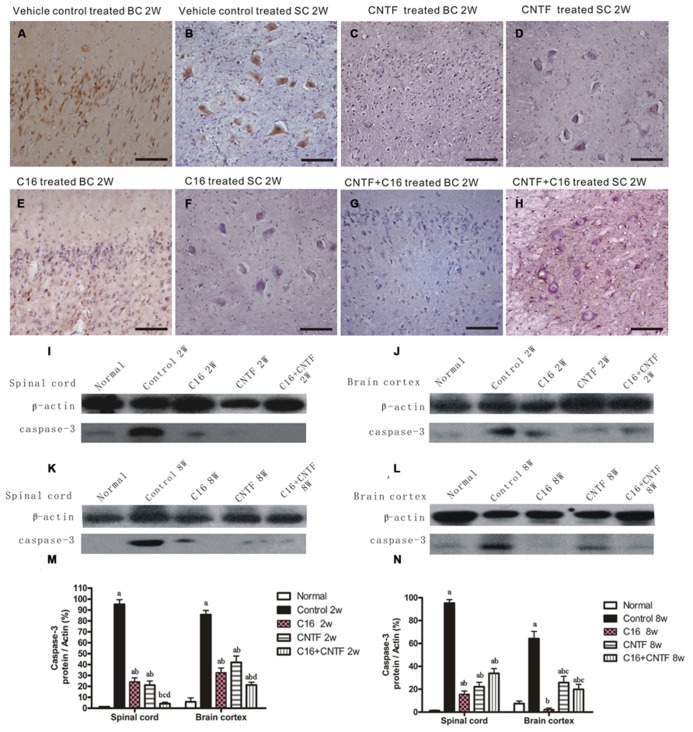
**Treatment with CNTF or C16 reduced the number of apoptotic neural cells in the brain cortex and spinal cord.** **(A–H)** Caspase-3 immunostaining, counterstained with hematoxylin, bar = 100 μm. [**(A,C,E,G)**: coronal sections of brain motor cortex; **(B,D,F,H)**: traverse sections through anterior horn of lumbar spinal cord]. At week 2 post-immunization, vehicle-treated EAE rats **(A)**: many caspase-3 labeled apoptotic neural cells appeared in the hindlimb area of the motor cortex; **(B)** plenty of caspase-3 labeled apoptotic neural cells appeared in the anterior horn of the spinal cord). In CNTF- **(C,D)**, C16- **(E,F)**, and C16+CNTF-treated **(G,H)** rats, the density of caspase-3 expression in neuronal cells was decreased, and the number of caspase-3+ apoptotic neural cells was also obviously reduced. Caspase-3 expression declined markedly both in the spinal cord **(I,K)** and cerebral cortex **(J,L)** following treatment with CNTF or C16 at weeks 2 and 8 post-immunization, as shown by western blotting. **(M,N)** Caspase-3 expression in the spinal cord and brain cortex at week 2 **(M)** and week 8 **(N)** post-immunization revealed by western blotting. *^a^P* < 0.05 versus the normal control; *^b^P* < 0.05 versus vehicle-treated EAE rats; ^c^*P* < 0.05 versus C16-treated EAE rats; ^d^*P* < 0.05 versus CNTF-treated EAE rats.

Western blot analysis also showed the expression of apoptotic signal molecule caspase-3 (an enzyme involved in the execution of the mammalian apoptotic cell death program) evidently increased in the vehicle control, and this effect was significantly reversed by treatment with C16, CNTF, or C16 plus CNTF at both weeks 2 and 8 post-immunization (**Figure 8**). The change in the number of caspase-3 immunoreactive neuronal cells in each group was consistent with the expression levels detected by Western blotting (**Figures 8A–H**).

### EFFECTS OF TREATMENT WITH CNTF AND/OR C16 ON REACTIVE ASTROCYTE PROLIFERATION AND REACTIVE GLIOSIS IN EAE RATS

**FIGURE 9:**
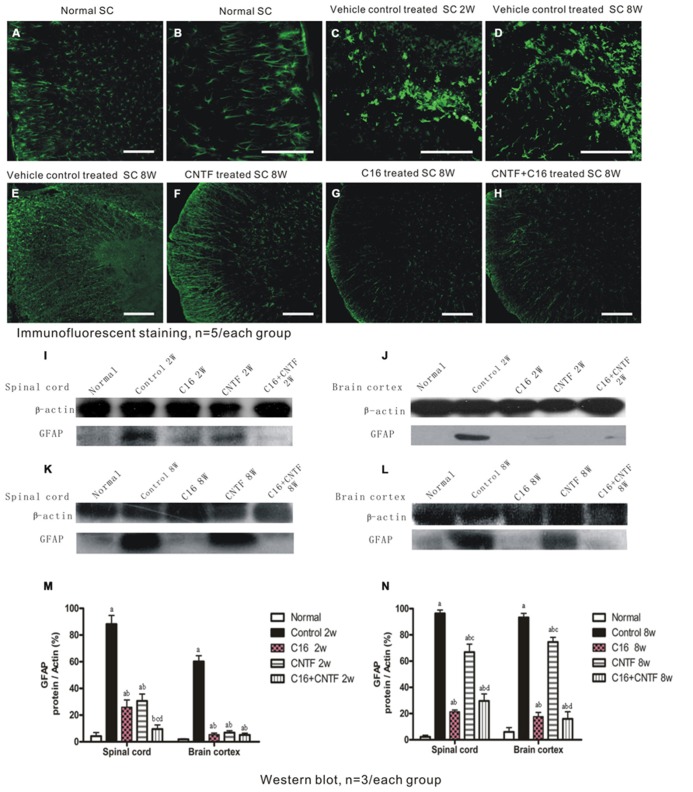
**Treatment with CNTF or C16 inhibited reactive gliosis, as revealed by FITC-conjugated GFAP immunofluorescent staining (traverse sections through the lumbar spinal cord), bar = 100 μm.** At week 2 post-immunization; **(A)** normal control group, **(B)** enlarged image showed the normal astrocytes. **(C,D)** Vehicle-treated EAE rats, showing astrocyte proliferation and formation of a visible glial scar (in **(C)**]. At week 8 post-immunization, vehicle-treated EAE rats **(E)** and CNTF-treated EAE rats **(F)** all exhibited evident glial scar formation, while reactive gliosis markedly decreased in C16- **(G)** and C16+CNTF-treated **(H)** EAE rats. **(I-L)** GFAP expression was visibly reduced at week 2 post-immunization following CNTF, C16, and C16+CNTF treatment, but not at week 8 post-immunization following CNTF treatment, as shown by Western blotting. **(M,N)**: *^a^P* < 0.05 versus normal control; *^b^P* < 0.05 versus vehicle-treated EAE rats; *^c^P* < 0.05 versus C16-treated EAE rats; *^d^P* < 0.05 versus CNTF-treated EAE rats.

To assess the effects of treatment with CNTF and C16 on EAE-induced reactive gliosis, we examined the expression of GFAP, a marker for astrocytes, by Western blot analysis and immunofluorescence labeling. Immunolabeling showed that astrocytes proliferated and formed a visible glial scar (**Figures 9C,D**). Westerns blot revealed that the expression of GFAP increased in both the spinal cord and cerebral cortex of vehicle-treated EAE rats (**Figures 9I–N**). Additionally, GFAP expression and astrocyte proliferation were significantly reduced in both C16- and CNTF-treated group at week 2 post-immunization (**Figure 9**), and the combined treatment with C16 plus CNTF resulted in an even better suppressing effect on glial scar formation (**Figure 9**). However, at week 8 post-immunization, the C16- and C16 plus CNTF-treated groups exhibited a more marked inhibition of astrocytes proliferation than the CNTF-treated group (**Figures 9G–H**). GFAP expression in the CNTF-treated group was obviously increased and approached the observed GFAP expression of the vehicle control at this time point (**Figure 9F**).

### EFFECTS OF TREATMENT WITH CNTF AND C16 ON THE EXPRESSION OF THE PRO-INFLAMMATORY CYTOKINES TNF-α AND IFN-γ AND THE ANTI-INFLAMMATORY CYTOKINE TGF-β

**FIGURE 10:**
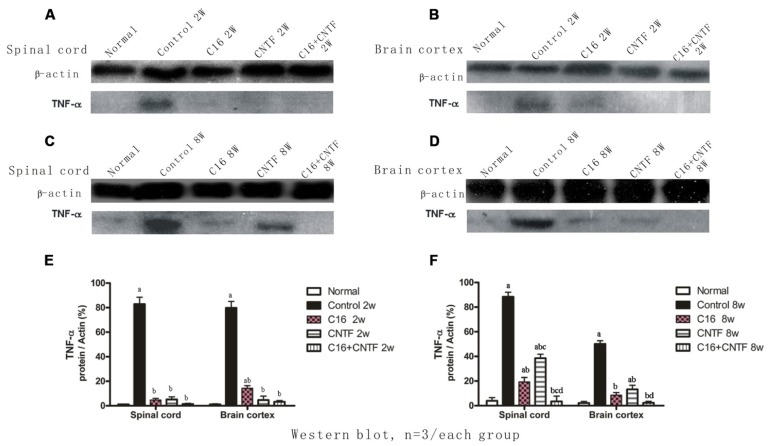
**TNF-α expression decreased markedly in both the spinal cord (A,C) and cerebral cortex **(B,D)** following treatment with CNTF or C16, as compared with the vehicle control, at weeks 2 and 8 post-immunization, as shown by Western blotting.** **(E,F)** TNF-α expression in the spinal cord and brain cortex at week 2 **(E)** and week 8 **(F)** postimmunization revealed by Western blotting. *^a^P* < 0.05 versus normal control; *^b^P* < 0.05 versus vehicle-treated EAE rats; *^c^P* < 0.05 versus C16-treated EAE rats; *^d^P* < 0.05 versus CNTF-treated EAE rats.

The results of Western blot analysis showed that at early (week 2) and late (week 8) stages of the clinical course, the motor cortex and spinal cord of vehicle control-treated EAE rats contained high levels of TNF-α expression (**Figure 10**). Also, a marked decrease in TNF-α expression in C16-, CNTF-, and C16 plus CNTF-treated groups, from weeks 2 to 8 post-immunization was confirmed by Western blotting (**Figure 10**).

**FIGURE 11:**
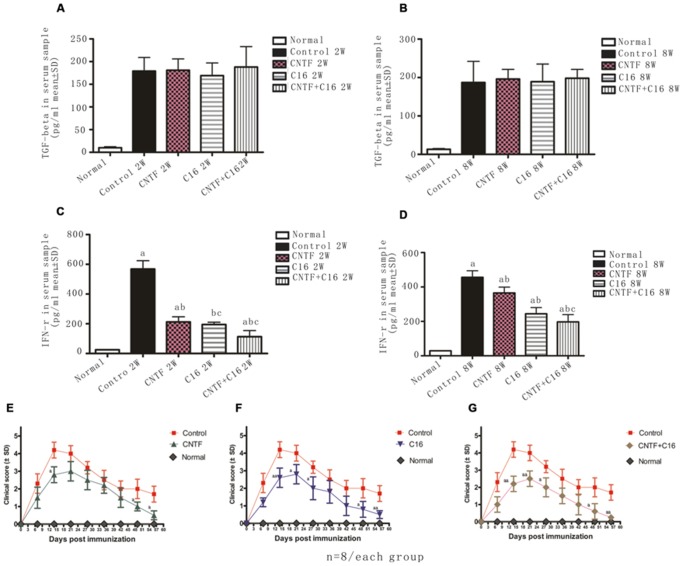
**(A–D)** Treatment with CNTF and C16 reduced the expression of the pro-inflammatory cytokines IFN-γ but no evident effects on the expression of anti-inflammatory cytokine TGF-β. **(A,B)** TGF-b levels in plasma samples from different groups at weeks 2 **(A)** and 8 **(B)** post-immunization, as measured by ELISA. **(C,D)** IFN-γ levels in plasma samples from different groups at weeks 2 **(A)** and 8 **(D)** post-immunization, as measured by ELISA. *^a^P* < 0.05 versus the normal control; *^b^P* < 0.05 versus vehicle-treated EAE rats; *^c^P* < 0.05 versus CNTF-treated EAE rats. **(E–G)** Treatment with CNTF or C16 halted disease progression and reduced disease severity. The clinical progression of EAE was attenuated after CNTF **(E)**, C16 **(F)**, and C16+CNTF **(G)** treatments. *^a^P* < 0.05 versus the vehicle-treated group at the same time point. *^aa^P* < 0.01 versus the vehicle-treated group at the same time point.

Next, the expression of IFN-γ and TGF-β in blood serum was measured by enzyme-linked immunosorbent assay (ELISA). Results showed that the expression of TGF-β in C16-, CNTF-, and C16 plus CNTF-treated groups all increased when compared with that in the normal control, but did not show significant improvement when compared with that in vehicle-treated EAE rats (**Figures 11A,B**). Nevertheless, treatment with C16, CNTF, or C16 plus CNTF all noticeably reduced the expression of IFN-γ, which was obviously increased in the blood serum of vehicle control-treated EAE rats (**Figures 11C,D**).

### EFFECTS OF TREATMENT WITH CNTF AND C16 ON DISEASE PROGRESSION AND SEVERITY

In vehicle-treated rats, disease symptoms appeared on day 7–9 post immunization. The acute phase of the disease began with a sharp increase in motor symptoms, which peaked at week 2 post-immunization (**Figures 11E–G**). Thereafter, the clinical score gradually declined. At week 8 post-immunization, the clinical score of surviving vehicle-treated animals returned to a level of 1–2 (**Figures 11E–G**). In general, animals treated with C16, CNTF, or C16 plus CNTF showed a similar disease course as the vehicle group, with a delayed onset (when the clinic score exceeded 2, on day 9–12 post-immunization), an obviously reduced clinical score in peak stage and a more rapid recovery. All of these effects were especially notable in the combined C16 plus CNTF-treated group (**Figures 11E–G**).

## DISCUSSION

Multiple sclerosis is characterized by inflammation, demyelination, axonal injury, and neuronal loss (Noseworthy et al., 2000; Hellings et al., 2002; El Behi et al., 2005). Hallmarks of active MS include perivascular infiltrates of microglia-derived macrophages, activated T lymphocytes, and reactive changes of astrocytes (Makar et al., 2008). Tissue damage and demyelination are caused by macrophages and T lymphocytes, which secrete pro-inflammatory cytokines, such as interleukin-1 (IL-1), IFN-γ, and TNF-α (Zindler and Zipp, 2010; Hou et al., 2012; Yin et al., 2012). This extensive neuroinflammation results in ongoing demyelination and axonal/neuronal injury mirrored by progressive physical disability (Tsutsui et al., 2004). Several immunomodulatory agents that inhibit this neuroinflammation have been shown to influence the course of MS (Kerschensteiner et al., 2004; Stanislaus et al., 2004). Thus, regulating inflammatory mediators and reducing innate immune activation is a rational therapeutic strategy for the treatment of MS.

Experimental autoimmune encephalomyelitis is still the most widely accepted animal model of MS. Different types of EAE have been developed in order to investigate pathogenetic, clinical, and therapeutic aspects of the heterogenic human disease. Generally, investigations in EAE are more suitable for the analysis of histopathological features (inflammation, demyelination, and degeneration) of the disease than for screening of new treatments. For example, even aggressive immunosuppression is not sufficient to treat progressive MS. All EAE models are directly accessible to investigation of the immune and nervous system, which interact during the pathogenesis of the disease and which are both targeted by established and experimental therapies. Despite extensive screening for new targets of MS therapy in EAE so far, only a few of the established MS therapies have been developed in the animal model (examples are glatiramer acetate, mitoxantrone, and natalizumab), all these therapies were set to target the pathogenesis of the disease process just like our agents (Mix et al., 2010).

In the current study, we showed that treatment with CNTF and/or C16 attenuated perivascular/parenchymal inflammation and decreased the size of lesions which was associated with the persistent inflammatory infiltration and perivascular edema. The inhibition of ανβ3 integrins has been shown to reduce leukocyte-endothelial interactions in a reperfusion model (Ichioka et al., 2007). The C16 peptide can recognize and bind to ανβ3, which plays an important role in leukocyte accumulation and adhesion processes (Weerasinghe et al., 1998; Ponce et al., 1999; Han et al., 2010), and competitively block transmigration of leukocytes attempting to cross the endothelium. In agreement with this hypothesis on the mechanism of C16 peptide, our previous studies demonstrated that the widespread perivascular and parenchymal infiltration of leukocytes, lymphocytes, and extravasated macrophages in the CNS of EAE rats were all significantly suppressed by consecutive IV injection of C16 (Fang et al., 2013). Inflammation scores were also clearly decreased compared with that of the vehicle control. Moreover, in addition to taking part in the infiltration process of inflammatory cells, ανβ3 integrin is thought to be an important receptor that regulates macrophage differentiation and macrophage responses to external signaling and maintains chronic inflammatory processes in pathological conditions (Antonov et al., 2011). Thus, besides interfering with the leukocyte transmigration, C16 application may also directly inhibit macrophage-related inflammation via blockage of ανβ3. While we gave daily injections for 2 weeks, half of the animals survived for 8 weeks. Thus, these sustained neuroprotective effects observed at the later stage of the EAE model (week 8 post-immunization) suggested that neurotrophic treatments may have lasting effects, even when treatment has been halted.

Previous studies of CNTF have demonstrated that it impairs inflammation in mouse models of MS (Stankoff et al., 2002; Kuhlmann et al., 2006). Moreover, in the acute phase of EAE, treatment with CNTF does not change the peripheral immune response, but did reduce the number of perivascular infiltrates of T cells and the levels of diffuse microglial activation in the spinal cord (Kuhlmann et al., 2006). In addition, blood brain barrier (BBB) permeability is significantly reduced in CNTF-treated animals (Kuhlmann et al., 2006). It is speculated that the clinical benefits of CNTF in EAE are caused mainly by preventing inflammatory cell recruitment to the CNS via effects on the BBB (Kuhlmann et al., 2006), and this mechanism overlaps, at least in part, with the proposed mechanism of C16. Our results showed that at the early stage of disease progression (week 2 following immunization), CNTF treatment markedly suppressed the infiltration of inflammatory cells, in accordance with the above-mentioned speculation on CNTF. However, at later stages (week 8 following immunization), CNTF administration did not cause an obvious reduction in inflammation, this phenomenon was consistent with the non-lasting anti-inflammatory effects of CNTF, which has been previously reported by Kuhlmann et al. (2006).

Widespread demyelination and axonal loss are the pathological hallmarks of MS (Pluchino et al., 2003). Microglial activation and migration is essential for the development of demyelination (Pluchino et al., 2003). The loss of myelin-derived trophic support caused axonal damage and the primary inflammatory attack on axonal targets, which is also associated with acute motor axon neuropathy (Bjartmar et al., 1999; Bannerman et al., 2005; Huizinga et al., 2008). The inflammation, demyelination, and axonal fragmentation in the CNS and PNS lead to motor neuron injury, which further leads to atrophy and neuronal death (Huizinga et al., 2008; Dutta and Trapp, 2011). Motor neuronal abnormalities contribute to spinal cord atrophy, limb weakness, and physical disability (Bjartmar et al., 1999; Huizinga et al., 2008; Dutta and Trapp, 2011). Additionally, neurological recovery in acute EAE rats has also been shown to be associated with the restoration of nerve conduction in the CNS and PNS via remyelination of demyelinated fibers by oligodendrocytes or Schwann cells (Pender, 1989). Besides demyelination, survival and differentiation of neurons also contributes to disease progression. The role of CNTF as a survival and differentiation factor for neurons and oligodendrocytes is well established (Clatterbuck et al., 1993; Linker et al., 2002). Previous studies have shown that CNTF promotes the survival of neurons and reduces serum deprivation-induced cell death of oligodendrocytes (Kuhlmann et al., 2006). In myelin oligodendrocyte glycoprotein-induced EAE, the disease was more severe and the vacuolar dystrophy of myelin, axonal damage, and neuronal loss were also more serious in CNTF-deficient mice than in wild-type mice (Linker et al., 2002). Moreover, CNTF acts on oligodendrocytes by favoring their final maturation and promoting remyelination in MS (Stankoff et al., 2002). All of these studies implied the direct neuroprotective effects of CNTF in the inflammatory environment and are consistent with our results of CNTF treatment, which showed that CNTF administration could alleviate demyelination, prevent axon loss, and reduce neuronal death. Even at a later stage, when inflammation returned after cessation of CNTF treatment, many more intact myelin, axons, and neurons could still be detected.

In acute EAE models, infiltrated leukocytes and activated macrophages/microglia may produce a pro-inflammatory milieu, strip off myelin from axons, and induce apoptosis in neurons and oligodendrocytes that are highly vulnerable to an aggravated microenvironment (Crowe et al., 1997; Liu et al., 1997; Warden et al., 2001). Therefore, targeting the neuroinflammatory reaction has been critical in the protection of motor neurons and the alleviation of clinical motor symptoms. In our study, C16 treatment resulted in a decrease in demyelination and relief of axonal damage at both weeks 2 and 8 post-immunization. The less abnormal ultrastructure and the more remyelinated axons observed under electron microscopy further confirmed the positive effects of C16 treatment on myelin and axons. All these phenomena may be ascribed to the amelioration of the inflammatory milieu. The improved microenvironment could further reduce the death of neurons and oligodendrocytes, which would be beneficial for the remyelination of demyelinated fibers.

Reactivated astroglia, which accumulated within and at the margins of demyelinating lesions, were GFAP-positive cells that were substantially larger than normal stellate astroglia. These cells are derived from adult radial glias that undergo mitosis and phenotypic transformation soon after the onset of CNS inflammation (Bannerman et al., 2007). The phenomenon of astrogliosis is due to both astroglial hyperplasia and hypertrophy in regions proximal to inflammatory infiltrates (Bannerman et al., 2007). Previous studies have shown that the migration of oligodendroglial lineage cells into lesions was retarded by intense perilesional gliosis (Bannerman et al., 2007). Reactive astroglia also contributed to the inflammatory response in MS and EAE by synthesizing pro-inflammatory cytokines and chemokines and by presenting peptide antigens to T lymphocytes (Balasingam et al., 1994). Diminishing reactive astrogliosis in and around EAE and MS lesions could diminish oligodendroglial death, enhance oligodendroglial regeneration, and facilitate remyelination (Bannerman et al., 2007). Our results indicated that C16 and CNTF treatment could reduce the proliferation of reactive astrocytes and decrease reactive gliosis, which may contribute to their beneficial effects at impairing inflammation. With the improved regional microenvironment and decreased demyelination, C16 treatment caused an obvious suppression of reactive astrocyte proliferation. The effects of CNTF on reactive astrogliosis were similar to those of C16 at week 2 post-immunization, which was consistent with its anti-inflammatory effects when the drug was delivery persistently. Nevertheless, after cessation of CNTF treatment, GFAP+-reactive astroglia and GFAP expression were increased in the CNTF-treated group, which was possibly related to the return of the inflammatory response and the facilitative effects of CNTF on the survival of glial cells.

Microglia, macrophages, and astrocytes have been implicated in the pathogenesis of MS by secreting a number of molecules, such as the pro-inflammatory cytokines TNF-α, IL-6, and IFN-γ, which act as inflammatory mediators and/or tissue damaging agents Stanislaus et al., 2002) CD4+ T helper 1 (Th1) lymphocytes can also produce TNF-α and IFN-γ, which could further induce a local influx of inflammatory cells and worsen EAE disease progression (Garay et al., 2007). The administration of CNTF and C16 can block perivascular infiltrates of inflammatory cells, alleviate microglial/macrophage activation, and inhibit reactive astrocyte proliferation; thus, pro-inflammatory factors produced by these cells would decrease accordingly. Moreover, previous studies have suggested that CNTF may counterbalance this effect of TNF-α, which further inferred the downstream signal transduction pathway and reduced the secondary transmigration of inflammatory cells (Linker et al., 2002); this may explain how the combined treatment further decreased the levels of TNF-α and IFN-γ.

On the other hand, the expression of TGF-β, an anti-inflammatory cytokine produced by CD4+ T helper 2 (Th2) cells, increased slightly over that of the normal control in vehicle-, CNTF-, C16-, and C16+CNTF-treated EAE rats (Zhang et al., 2006). This phenomenon may be a compensation reaction in the inflammatory microenvironment. However, the expression of TGF-β in C16-, CNTF-, and combined C16+CNTF-treated groups showed no significant improvement when compared with vehicle-treated EAE rats, which may be due to similar suppressing effects on Th1 and Th2 cells.

Previous and recent results demonstrated that C16 could inhibit the extensive infiltration of leukocytes and activation of macrophages by competitively blocking the integrins interfering with the leukocyte transmigration. Moreover, by improving the microenvironment, it may alleviate disease severity at the peak time of EAE progression and lead to a more rapid recovery of locomotor function. In addition to impaired inflammation, CNTF also exerted direct neuroprotective effects, alleviating demyelination, preventing axon loss, and reducing neuronal death, which contributed to the delay of disease onset and the decline of maximum clinical scores. Compared with individually treatment, the combined treatment with C16 and CNTF produced more dramatic effects on the inflammation inhibition, neuroprotection, and functional recovery. These results suggested that, even though they have partly overlapping underlying mechanisms, CNTF and C16 still could exert their neuro-protective actions and gain better synergistic effects through targeting different pathways.

## AUTHOR CONTRIBUTIONS

Shu Han designed the experiments and drafted the manuscript, Marong Fang and Hong Jiang participated in the study design and coordination, DaQiang He and Jing Yang performed the experiments, Fan Zhang and Zhiying Hu analyzed the data and revised the manuscript. All authors read and approved the final manuscript.

## Conflict of Interest Statement

The authors declare that the research was conducted in the absence of any commercial or financial relationships that could be construed as a potential conflict of interest. The Reviewer Dr. Yew David Tai Wai declares that, despite having collaborated with Dr. Marong Fang in the past, the review process was handled objectively and no conflict of interest exists.

## ABBREVIATIONS

ABC: avidin–biotin peroxidase complex
ANOVA: analysis of variance
BBB: blood-brain barrier
CFA: complete Freud adjuvant
CNS: central nervous system
CNTF: ciliary neurotrophic factor
EAE: experimental allergic encephalomyelitis
GFAP: glia fibrillary acidic protein
GPSCH: guinea pig spinal cord homogenate
ICAM-1: intercellular adhesion molecule-1
IFN-γ: interferon-γ
MRI: magnetic resonance imaging
MS: multiple sclerosis
PNS: peripheral nerve system
TEM: transendothelial migration
TGF-β: transforming growth factor beta
TNF-α: tumor necrosis factor-α
